# Comparison of Bootstrap Methods for Estimating Causality in Linear Dynamic Systems: A Review

**DOI:** 10.3390/e25071070

**Published:** 2023-07-17

**Authors:** Fumikazu Miwakeichi, Andreas Galka

**Affiliations:** 1 Department of Statistical Modeling, The Institute of Statistical Mathematics, Tokyo 190-8562, Japan; 2 Statistical Science Program, Graduate Institute for Advanced Studies, SOKENDAI, Tokyo 190-8562, Japan; 3 Clinic for Pediatric and Adolescent Medicine II, University Clinic, University of Kiel, 24105 Kiel, Germany; a.galka@pedneuro.uni-kiel.de

**Keywords:** causal analysis, Granger causality, bootstrap methods, multivariate time series, impulse response function

## Abstract

In this study, we present a thorough comparison of the performance of four different bootstrap methods for assessing the significance of causal analysis in time series data. For this purpose, multivariate simulated data are generated by a linear feedback system. The methods investigated are uncorrelated Phase Randomization Bootstrap (uPRB), which generates surrogate data with no cross-correlation between variables by randomizing the phase in the frequency domain; Time Shift Bootstrap (TSB), which generates surrogate data by randomizing the phase in the time domain; Stationary Bootstrap (SB), which calculates standard errors and constructs confidence regions for weakly dependent stationary observations; and AR-Sieve Bootstrap (ARSB), a resampling method based on AutoRegressive (AR) models that approximates the underlying data-generating process. The uPRB method accurately identifies variable interactions but fails to detect self-feedback in some variables. The TSB method, despite performing worse than uPRB, is unable to detect feedback between certain variables. The SB method gives consistent causality results, although its ability to detect self-feedback decreases, as the mean block width increases. The ARSB method shows superior performance, accurately detecting both self-feedback and causality across all variables. Regarding the analysis of the Impulse Response Function (IRF), only the ARSB method succeeds in detecting both self-feedback and causality in all variables, aligning well with the connectivity diagram. Other methods, however, show considerable variations in detection performance, with some detecting false positives and others only detecting self-feedback.

## 1. Introduction

In many fields of scientific inquiry, the analysis of causal relationships in complex systems represents an important task, which depends on the synergistic interplay between theory and application. An important contribution in this area has been the proposal of Granger Causality (GC) [1], which tests the significance of dynamic influence between pairwise time series. Building upon this foundation, Geweke [2] expanded GC to encompass multivariate time series, introducing conditional Granger Causality (cGC). Bressler et al. [3] and Schiatti et al. [4] further refined cGC, enabling the estimation of causality among multivariate time series using Vector AutoRegressive (VAR) models.

Granger causality and its generalizations represent a statistical method that depends on the comparison of the residual variances resulting from applying different models to the same time series; a wide array of predictive models, including non-linear variants, may be employed. As an alternative approach to quantifying causality from predictive modeling, Impulse Response Function (IRF) analysis has been developed as a tool for examining variable responses to shocks within an identified model.

However, it is worth noting that Granger causality and IRF analysis always depend on the particular chosen model classes; they are not suitable for model discovery since they are lacking invariance theorems, as they exist for the more general concept of algorithmic complexity [5].

Conceptually, the statistical significance of numerically calculated cGC and IRF should be assessed using the asymptotic method. However, real-world data analysis may yield incorrect results due to potential distribution discrepancies. In cases where the distribution function is unknown, such as with partial Granger causality [6], the asymptotic method is inapplicable. The bootstrap strategy serves as a widely used alternative for cGC and IRF significance testing. However, with numerous bootstrap methods available, each with varying sensitivity and specificity, selecting the appropriate method remains a critical challenge when applying time series models to real data analysis.

In a causal analysis of time series data, failing to apply an appropriate bootstrap method can lead to several important issues. First, as causal analysis is based on the estimation of causal relationships, not using a suitable bootstrap method could potentially result in inaccuracies in estimating these relationships, thereby undermining the reliability of statistical inferences. Furthermore, bootstrap methods are often used for estimating confidence intervals, and if an unsuitable method is chosen, the confidence intervals could be under- or over-estimated, leading to inappropriate assessments of uncertainty. Additionally, bootstrap methods are used to estimate the magnitude of causal effects, and without a suitable method, the magnitude of these effects could be inaccurately estimated, which could affect the appropriateness of subsequent actions. Therefore, to avoid these problems, it is important to select and apply suitable bootstrap methods for causal analysis and choose methods based on understanding the characteristics of the time series data and the objectives of the causal analysis.

In this study, we thoroughly evaluate the performance of four bootstrap methods designed for time series analysis. The first method we present is an adaptation of the Phase Randomize Bootstrap (FRB), which generates surrogate data for hypothesis testing through phase randomization in the Fourier transform of original time series data. We propose a modification, the uncorrelated Phase-Randomized Bootstrap (uPRB), which randomizes phases for each variable in the spectral domain, for causality detection among variables. As a counterpart, we consider a technique based on Circular Block Bootstrap (CBB), a method for transfer randomization in the time domain [7]. This approach remedies the under-representation of the beginning and the end of the time series in surrogate data by creating a circular time series. Time-Shifted Surrogates (TSS) [8,9], another technique we discuss, ensures the preservation of all characteristics of the initial signal by maintaining the same stat–space trajectory. The Stationary Bootstrap (SB) [10] uses random block lengths for standard error calculations and confidence region construction in weakly dependent stationary observations. Last, we discuss the AR-Sieve Bootstrap (ARSB), a method that approximates the underlying data-generating process by fitting an AR model, useful for handling dependent data [11,12].

## 2. Causal Analysis

### 2.1. Conditional Granger Causality (cGC)

Let $Y_{t}={\left\{y_{t}^{1},\ldots ,y_{t}^{M}\right\}}^{T}$ denote the vector variables at time *t*, 1≤t≤N, where *N* denotes the length of the time series. The feedback system among the variables $Y_{t}$ can be represented by a basic Vector AutoRegressive (VAR) model of order *p*, defined as

(1) $$\begin{array}{c} Y_{t}=\sum_{k=1}^{p}A_{k}Y_{t-k}+E_{t}, \end{array}$$

where $A_{k}$ denotes a set of M×M-dimensional coefficient matrices, and $E_{t}={\left\{e_{t}^{1},\ldots ,e_{t}^{M}\right\}}^{T}$ denotes a series of shocks (disturbances), given by white noise vectors with zero means.

Extracting the *l*th variable of the VAR model gives

(2) $$y_{t}^{l}=\sum_{k=1}^{p}A_{k}^{l}Y_{t-k}+e_{t}^{l},$$

where $A_{k}^{l}=\left\{a_{k}^{l1},a_{k}^{l2},\ldots ,a_{k}^{lm},\ldots ,a_{k}^{lM}\right\}$ denotes the *l*th row of $A_{k}$. Equation (2) represents an autoregressive model with exogenous input (ARX model) that consists of an endogenous part of the *l*th variable and an exogenous part of all other variables.

The ARX model, excluding the *m*th variable, is called the restricted ARX (rARX) model:

(3) $$y_{t}^{l}=\sum_{k=1}^{p}{\tilde{A}}_{k}^{l}Y_{t-k}^{\left(m\right)}+e_{t}^{lm},$$

where $Y_{t}^{\left(m\right)}$ denotes the vector $Y_{t}$ with the *m*th element excluded (e.g., $Y_{t}^{\left(1\right)}={\left\{y_{t}^{2},\ldots ,y_{t}^{M}\right\}}^{T}$, $Y_{t}^{\left(2\right)}={\left\{y_{t}^{1},y_{t}^{3},\ldots ,y_{t}^{M}\right\}}^{T}$ and so on) and ${\tilde{A}}_{k}^{l}$ denotes a set of re-estimated (M−1)-dimensional coefficient vectors. Suppose that the past values of the *m*th variable $\left\{y_{t-1}^{m},y_{t-2}^{m},\ldots ,y_{t-p}^{m}\right\}$ contribute to the prediction of the *l*th variable $\left\{y_{t}^{l}\right\}$; then, the variances of the residuals should satisfy $\mathrm{var}\left(e_{t}^{l}\right)<\mathrm{var}\left(e_{t}^{lm}\right)$. The significance of the difference in variances can be evaluated by a likelihood ratio test:

(4) $$F_{m\to l}=\log\frac{\mathrm{var}\left(e_{t}^{lm}\right)}{\mathrm{var}\left(e_{t}^{l}\right)}.$$

The null hypothesis of absence of conditional Granger causality from the *m*th to the *l*th element given $Y_{t}^{m}=\left\{y_{1}^{m},y_{2}^{m},\ldots ,y_{p}^{m}\right\}$ states that all $a_{k}^{lm}$ were zero, i.e., $H_{0}:a_{1}^{lm}=a_{2}^{lm}=\ldots =a_{p}^{lm}=0$. This implies that past values of $Y_{t}^{m}$ do not improve the prediction of $y_{t}^{l}$. On the contrary, the rejection of this null hypothesis suggests that $Y_{t}^{m}$ in Granger causes $y_{t}^{l}$.

### 2.2. Impulse Response Function (IRF)

There is another representation of the VAR model of Equation (1) using a delay operator *L*, such that $LY_{t}=Y_{t-1}$:

(5) $$\begin{array}{ccc} A\left(L\right)Y_{t} & = & E_{t}, \end{array}$$

(6) $$\begin{array}{ccc} A(L) & = & I_{M}-A_{1}L-A_{2}L^{2}-\ldots -A_{p}L^{p}. \end{array}$$

where $I_{M}$ denotes the M×M-dimensional unity matrix. The roots (eigenvalues) $\lambda_{j}$ of the polynomial A(L) need to fulfill $\left|\lambda_{j}\right|<1$ for all *j*.

Further transformation of Equation (5) yields

(7) $$\begin{array}{ccc} Y_{t} & = & A{\left(L\right)}^{-1}E_{t}\\ & = & \left\{I_{M}+\Psi_{1}L+\Psi_{2}L^{2}+\ldots \right\}E_{t}. \end{array}$$

which demonstrates that a VAR model can be rewritten as

(8) $$Y_{t}=\sum_{k=0}^{\infty }\Psi_{k}E_{t-k}=\Psi \left(L\right)E_{t},$$

where

$$\begin{array}{ccc} \Psi (L) & = & A{\left(L\right)}^{-1}\\ & = & {\left\{I_{M}-A_{1}L-A_{2}L^{2}-\ldots -A_{p}L^{p}\right\}}^{-1}\\ & = & I_{M}+\Psi_{1}L+\Psi_{2}L^{2}+\ldots \end{array}$$

Equation (8) represents the Vector Moving Average (VMA) representation of the VAR model and can be denoted as VMA(*∞*).

Partial differentiation of the VMA(*∞*) model with respect to one particular shock (disturbance term) $E_{t}$ yields a set of derivatives

(9) $$\begin{array}{c} \frac{\partial Y_{t}}{\partial E_{t}}=I_{M},\;\frac{\partial Y_{t+1}}{\partial E_{t}}=\Psi_{1},\cdots ,\;\frac{\partial Y_{t+s}}{\partial E_{t}}=\Psi_{s}. \end{array}$$

The (l,m)th element of $\Psi_{s}$ is given by $\frac{\partial y_{t+s}^{l}}{\partial e_{t}^{m}}$, which represents the marginal effect (influence) from the *m*th shock $e_{t}^{m}$ to the *l*th variable $y_{t+s}^{l}$. The function defining the time series of such marginal effects is called the Impulse Response Function (IRF) and is denoted by ${\mathrm{IRF}}_{s}^{lm}$.

The null hypothesis for the IRF states that a one-time shock to the *m*th variable has no impact on future values of the *l*th variable. This can be represented as: $H_{0}:{\mathrm{IRF}}_{s}^{lm}=0$ for all s>0. The rejection of this null hypothesis suggests that a one-time shock to the *m*th variable significantly affects the *l*th variable at some future point in time.

## 3. Bootstrap Methods

### 3.1. Uncorrelated Phase Randomization Bootstrap (uPRB)

The Phase Randomization Bootstrap method has been proposed as a technique to generate surrogate time series data for hypothesis testing [13,14]. Using the Fourier transform, the method transforms the original time series data into the frequency domain. Then, the phase of each frequency component is randomized while preserving the original amplitude spectrum. Finally, the surrogate data are obtained by applying the inverse Fourier transform to the randomized frequency components.

This process generates surrogate time series data with the same power spectrum as the original data but with disrupted temporal correlations. By comparing the original data’s non-linearity measures or other statistical properties to those of the surrogate data, researchers can test hypotheses and detect the presence of non-linear dynamics in the original time series data. This method has been applied in various fields, including the study of physiological signals [15], economics and finance [16], climate data [9], and so on.

In order to preserve all linear auto-correlations and cross-correlations, the Phase Randomization Bootstrap adds a common random sequence φ(f) to the phases of all variables. Thus, since this method cannot detect causality among variables, we prepare random sequences independently for each variable. In this paper, we call this method uncorrelated Phase Randomization Bootstrap (uPRB).

The procedure for generating surrogate datasets by uPRB is as follows.

**Step** **1**  Transform the original time series by applying Fourier transform to each variable as
  $$Y^{l}\left(f\right)=F\left\{y_{t}^{l}\right\}=A^{l}\left(f\right)e^{i\phi^{l}\left(f\right)},$$
  where *l* is the index of the variable, and $A^{l}\left(f\right)$ and $\phi^{l}\left(f\right)$ denote the amplitude and the phase, respectively.
**Step** **2**  For each frequency *f*, add an independent random value $\varphi^{l}\left(f\right)$ following a uniform distribution throughout the interval [0,2π) to $\phi^{l}\left(f\right)$, while satisfying the symmetry property $\varphi^{l}\left(f\right)=-\varphi^{l}\left(-f\right)$. That is,
  $${\tilde{Y}}^{l}\left(f\right)=A^{l}\left(f\right)e^{i[\phi^{l}\left(f\right)+\varphi^{l}\left(f\right)]}.$$
**Step** **3**  Transform the spectral domain representation back to the time domain by applying the inverse Fourier transform to each variable as
  $$y_{t}^{*l}=F^{-1}\left\{{\tilde{Y}}^{l}\left(f\right)\right\}=F^{-1}\left\{Y^{l}\left(f\right)e^{i\varphi^{l}\left(f\right)}\right\}.$$
**Step** **4**  Repeat Steps 2–3 for all variables.
**Step** **5**  Repeat Steps 2–4 a large number of times, thereby generating a set of surrogate datasets.

### 3.2. Stationary Bootstrap (SB)

The conventional Non-overlapping Block Bootstrap (NBB) has been proposed by Carlstein [17]. By improving this conventional method, Künsch [18] has proposed a Moving-Blocks Bootstrap (MBB). This method is useful, especially for small sample data having a wider range of blocks than the conventional method. However, in the process of random sampling, there is an edge effect of the uneven weighting of the selection at the beginning and end of the data. To compensate for this shortcoming, Politis and Romano [7] proposed a Circular Block Bootstrap (CBB) that concatenates the start and end points of the original data. In the block bootstrap method, the stationarity of the sample is an important assumption. However, surrogate data obtained by resampling using the above-mentioned method are not necessarily stationary. Therefore, the Stationary Bootstrap (SB) method has been proposed, in which the surrogate data are also stationary. This method is similar to the TSS method, except that the block width is not fixed but is resampled as a random variable following a geometric distribution [10]. The procedure for generating surrogate data by SB is as follows.

**Step** **1**  Set the mean block width to *w*. Then, in the geometric distribution used in Step 3, we have p=1/w.
**Step** **2**  Duplicate the original data and merge it to the end of the original data such that $Y_{N+1}=Y_{1},Y_{N+2}=Y_{2},\ldots ,Y_{2N}=Y_{N}$.
**Step** **3**  Generate a sequence of natural random numbers $L_{1}^{*b},\ldots ,L_{K}^{*b}$ corresponding to each block width following a geometric distribution so that the probability of the event $L_{l}^{*b}=r$ is ${\left(1-p\right)}^{r-1}p$ for r=1,2,…. Here, the value of *K* is determined to satisfy the condition $K=\min\{k:\sum_{l=1}^{k}L_{l}^{*b}\geq N\}$.
**Step** **4**  Generate a sequence of natural random numbers $I_{1}^{*b},\ldots ,I_{K}^{*b}$, corresponding to the index of the starting point of the block, following a uniform distribution over the interval [1,N].
**Step** **5**  The blocks, $\left\{\xi^{*b}\left(I_{1}^{*b},L_{1}^{*b}\right),\ldots ,\xi^{*b}\left(I_{K}^{*b},L_{K}^{*b}\right)\right\}$, constructed according to Steps 1 and 2, are arranged in the order in which they were extracted, and a pair of resamples is obtained with the first *N* elements as $Y_{1}^{*b},\ldots ,Y_{N}^{*b}$.
**Step** **6**  Repeat Steps 3–5 a large number of times, thereby generating a set of surrogate datasets.

In the case of multivariate time series, there are two ways to perform Steps 3–5. One way is to use the same blocks $\left\{\xi^{*b}\left(I_{1}^{*b},L_{1}^{*b}\right),\ldots ,\xi^{*b}\left(I_{K}^{*b},L_{K}^{*b}\right)\right\}$ for all variables for data shuffling, as described above, and the other is to perform Steps 3–5 for each variable independently. In this paper, we refer to the former as correlated SB (cSB) and to the latter as uncorrelated SB (ucSB).

### 3.3. Time Shift Surrogates (TSS)

While PRB is a method for generating surrogate datasets by randomizing the phases in the frequency domain, we evaluated the performance of TSS as a method of randomizing the phase in the time domain. This method corresponds to the special case of the CBB, where the number of blocks is limited to 1. The procedure for generating surrogate datasets by TSS is as follows.

**Step** **1**  Duplicate the original data and merge it to the end of the original data such that $Y_{N+1}=Y_{1},Y_{N+2}=Y_{2},\ldots ,Y_{2N}=Y_{N}$.
**Step** **2**  Generate a natural random number *s* following a uniform distribution over the interval $\left[I_{a},I_{b}\right]$, extract a sequence of the data
  $\left\{y_{s}^{l},\ldots ,y_{N+s}^{l}\right\}$, and use it as surrogate dataset for the *l*th variable.
**Step** **3**  Repeat Step 2 for all variables.
**Step** **4**  Repeat Steps 2–3 a large number of times, thereby generating a set of surrogate datasets.

### 3.4. AR-Sieve Bootstrap (ARSB)

The AR-Sieve Bootstrap (ARSB) method generates surrogate datasets by feeding a VAR model, which employs re-estimated parameters, with residuals from modeling [11,12].

The procedure for generating surrogate datasets using ARSB is as follows.

**Step** **1**  Fit the VAR model of Equation (1) to the original time series, obtaining estimates for the parameters and the residuals ${\hat{E}}_{t}$.
**Step** **2**  Compute centered residuals ${\hat{E}}_{1}-\bar{E},\ldots ,{\hat{E}}_{N}-\bar{E}$, where
  $\bar{E}=N^{-1}\sum_{t=1}^{N}{\hat{E}}_{t}$, and generate bootstrap residuals $E_{1}^{*},\ldots ,E_{N}^{*}$ by shuffling the indices according to a sequence of natural random numbers $\left\{J_{1}^{*b},\ldots ,J_{N}^{*b}\right\}$, which was drawn randomly with replacement from a uniform distribution over the interval [1,N].
**Step** **3**  Compute the surrogate time series recursively by
  $$Y_{t}^{*}=\sum_{k=1}^{p}{\hat{A}}_{k}Y_{t-k}^{*}+E_{t}^{*}$$
  where $\left(Y_{1}^{*},\ldots ,Y_{p}^{*}\right)=\left(Y_{1},\ldots ,Y_{p}\right)$.
**Step** **4**  Re-estimate the VAR parameter matrices $A_{1},\ldots ,A_{p}$ based on the bootstrap time series.
**Step** **5**  Repeat Steps 2–4 a large number of times, thereby generating a set of surrogate datasets.

Similar to the SB method, there are two ways to process Step 2 in the ARSB method. One is to use the same sequence of random values $\left\{J_{1}^{*b},\ldots ,J_{N}^{*b}\right\}$ for all variables for data shuffling, as mentioned above, and the other is to perform Step 2 independently for each variable. In this paper, we refer to the former as correlated ARSB (cARSB) and to the latter as uncorrelated ARSB (ucARSB).

## 4. Simulation

**Figure 1:**
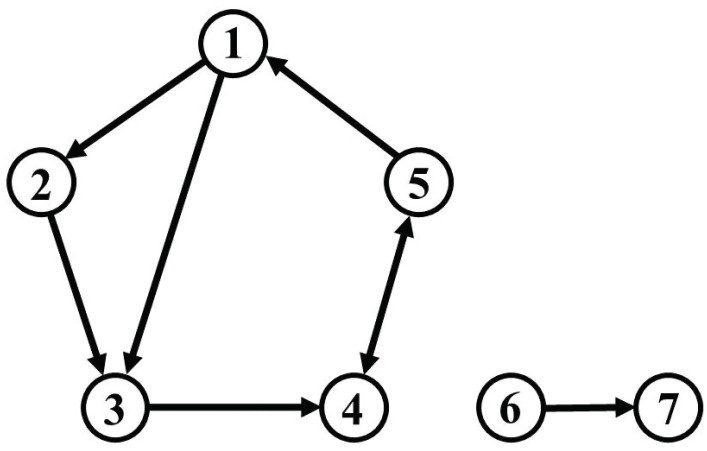
Connectivity diagram of the simulation model.

In order to verify the performance of the methods for causal analysis and of the bootstrap methods, as discussed above, we prepared a simulation model by modifying a model proposed by [19]. We set up seven oscillatory variables as depicted in Figure 1, five of which generated their stochastic oscillations locally from driving white noise via self-feedback; furthermore, the seven variables were coupled directly or indirectly within a global Vector AutoRegressive (VAR) process, such that a dynamic feedback system results. We fixed the frequency of each variable to 0.1 Hz and randomly selected a damping coefficient for each variable from a normal distribution. The sixth and seventh variables were isolated from the other five. For instance, the first and third variables were directly connected, while the first and fourth variables were indirectly connected through the third variable. The fourth and fifth variables were directly and bidirectionally connected.

The sixth variable generated a local oscillation at 0.1 Hz via self-feedback, like the other five, while the seventh variable did not have any self-feedback, but was driven only by the sixth variable. If causality would be detected between either the sixth or seventh variables and any of the first to fifth variables, this would represent a false positive.

We set the connectivity among variables as shown in Figure 1 and generate a simulated time series of length N=2000 through the VAR process of Equation (1), with p=2. The nominal sampling frequency was 10 Hz, and the series of shocks (disturbances) $E_{t}$ was sampled from a 7-dimensional multivariate Gaussian noise distribution with zero mean and diagonal covariance matrix given by $\Sigma_{E}=0.1I_{7}$.

**Figure 2:**
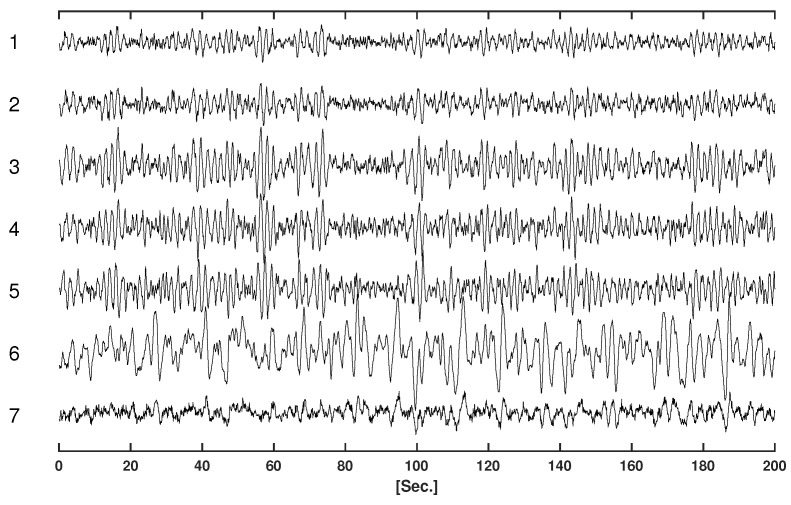
Simulated time series.

Table 1 displays the parameters of the VAR model for the simulations. Figure 2 displays the simulated time series.

## 5. Results

**Figure 3:**
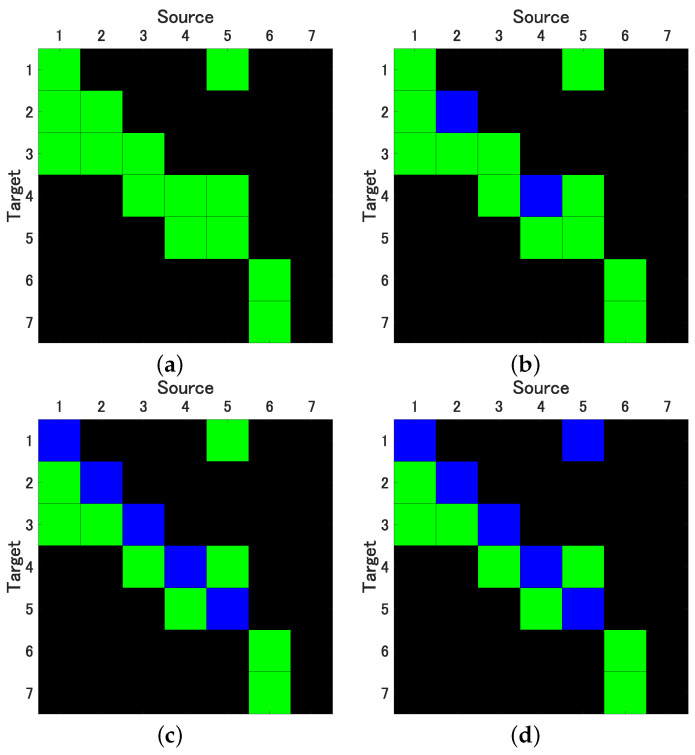
Results of evaluation of Granger Causality by correlated Stationary Bootstrap (cSB) with mean block width w=5 (**a**), and uncorrelated Stationary Bootstrap (ucSB) with mean block width w=10 (**b**) and w=40 (**c**), and by Time Shift Surrogates (TSS) (**d**); variables in columns represent sources, variables in rows represent targets (p<0.01); green: true positives, blue: false negatives.

In our pursuit of accurately estimating the 99% confidence intervals (CI) for both conditional Granger Causality (cGC) and Impulse Response Function (IRF) analyses, we generated 2000 surrogate datasets using each of the previously discussed bootstrap methods. For the SB method, we determined the optimal mean block width *w*, selecting values of 5, 10, 20, and 40. As illustrated in Figure 3a, the cSB method consistently yielded correct causality results, irrespective of the chosen mean block width. In contrast, the ucSB method produced correct causality results for w=5, but the detection rate of self-feedback diminished as *w* increased (see Figure 3b,c).

While the uPRB method provided accurate causality results concerning variable interactions, it failed to detect the self-feedbacks of the first through fifth variables, yielding results akin to ucSB(20) and ucSB(40) (see Figure 3c). If the time shift *s* in the TSS method was excessively small or large, the data closely resembled the original data, consequently minimizing the phase randomization effect. The selection of interval $\left[I_{a},I_{b}\right]$ in the Time Shift Surrogates (TSS) method has potential implications on the statistical characteristics of the generated surrogate data, particularly its correlation structure. For causality detection between variables, it is necessary to set the interval large enough beyond the lag where cross-correlation occurs. In this simulation, we set $\left[I_{a},I_{b}\right]=\left[3/N,0.9N\right]$.

Notably, the TSS method’s detection performance is inferior to that of the ucSB(20) and ucSB(40) methods, failing to detect causality from the fifth to the first variable. Importantly, our study identified no false positives within any bootstrap method. Table 2 concisely summarizes the sensitivity and specificity for each combination of causal analysis and bootstrap methods.

**Figure 4:**
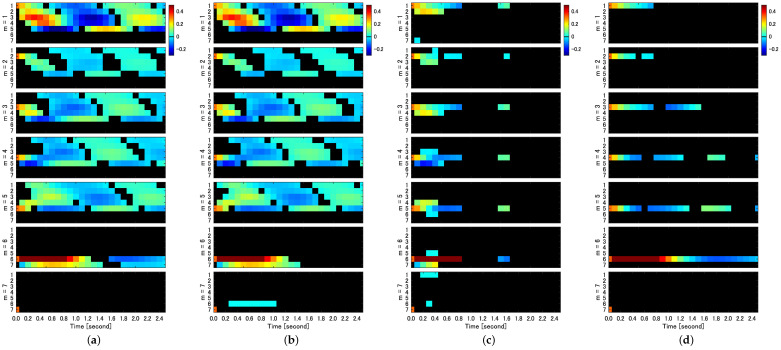
Significant impulse response function (${\mathrm{IRF}}_{s}^{lm}$) evaluated by uncorrelated AR-Sieve Bootstrap (ucARSB) (**a**), correlated Stationary Bootstrap (cSB) with mean block width w=40 (**b**), uncorrelated Phase Randomization Bootstrap (uPRB) (**c**), and uncorrelated Stationary Bootstrap (ucSB) with mean block width w=40 (**d**). Responses exceeding the significance level (p<0.01) are displayed using color coding (see colorbars). Non-significant parts of impulse response functions remain black. Each column of subfigures displays ${\mathrm{IRF}}_{s}^{lm}$ for m=1,…,7 (subfigures from top to bottom) and for each value of *m* for l=1,…,7 (rows within each subfigure).

As shown in Equation (9), the IRF is denoted as ${\mathrm{IRF}}_{s}^{lm}$, which represents the impact of the *m*th shock $e_{t}^{m}$ on the *l*th variable $y_{t+s}^{l}$ at time *s*. We evaluated the significance of ${\mathrm{IRF}}_{s}^{lm}$ using each bootstrap method. Figure 4 provides a two-dimensional representation of significant ${\mathrm{IRF}}_{s}^{lm}$.

In the IRF analysis, only the ARSB method, whether correlated or uncorrelated, successfully detects both self-feedback and causality among variables in their entirety. Figure 4a displays the IRFs for the impact assigned to each variable (m=1,⋯,7), with ucARSB evaluating the significance. This outcome aligns with the connectivity diagram depicted in Figure 1. The cSB method demonstrates results were almost identical to the ARSB method for the first through fifth variables. However, it detects a response from the sixth to seventh variable, constituting a false positive, which was observed for all tested values of the mean block width. The uPRB method primarily detects causality between directly coupled variables (see Figure 4c). Additionally, false positives emerge where responses occur between uncoupled variables, such as the reciprocal response between the first and seventh variables and between the fifth and sixth variables. For all tested values of the mean block width, the ucSB method exhibits the weakest detection performance among the evaluated bootstrap methods, detecting only self-feedback (see Figure 4d).

To summarize the results of these IRFs, such as GC, we compared the true IRFs generated through the VAR parameters in Table 1 with the IRFs detected by the respective bootstrap methods and summarized the sensitivity and specificity in Table 2. The time interval of the IRFs used for comparison was the maximum time step before feedback occurs, i.e., the IRFs up to three time steps before the impact on the third variable propagates to the first variable to the fourth and fifth variables (see Figure 1).

## 6. Discussion

In this study, we present a thorough comparison of the performance of four different bootstrap methods (encompassing twelve variations, including different values of mean block width, as well as correlated and uncorrelated derivations) to evaluate the results from causal analyses.

**Figure 5:**
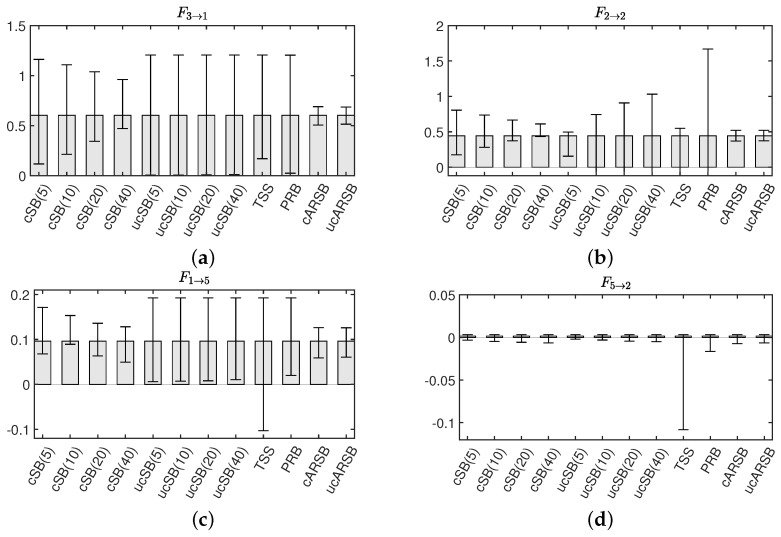
Representative examples of 99% confidence intervals of Granger Causality $F_{l\to m}$, estimated for each bootstrap method.

Our findings reveal that, for the cSB method, the Confidence Interval (CI) width decreases as the mean block width increases, which is particularly evident in Figure 5a,b. A possible explanation is that a shorter block width introduces more discontinuities in the surrogate time series, causing the dynamic properties to deviate further from those of the original time series due to the random shuffling of blocks disrupting temporal forward/backward relationships. This disruption may lead to falsely detecting causality from the seventh to sixth variables in the IRF analysis, as seen in Figure 4b. In contrast, the CI width for the ucSB method remains wider than that of the cSB method, irrespective of the mean block width (see Figure 5a,c), or even increases with the mean block width (Figure 5b). This stems from each block’s starting point being determined independently for each variable, causing unnecessary destruction of causality among variables in the surrogate time series. Consequently, no causality among variables is detected in the corresponding IRF analysis (see Figure 4d).

The TSS method accurately detects significance for the Granger causality $F_{1\to 3}$, but the CI widths for $F_{5\to 1}$ and $F_{2\to 2}$ are so large that they include zero, resulting in false negatives. This could be attributed to the (1,5)th element of $A_{2}$ having the smallest value among the VAR parameters corresponding to causality among variables, and the (2,2)th element of $A_{2}$ having the smallest value among the VAR parameters corresponding to the attenuation rate of self-feedback. A prime example is $F_{2\to 5}$, which should be numerically ignorable since our simulation model does not contain causality from the second to fifth variable (see Table 1). Although the TSS method correctly assesses non-significance, the CI width is substantially larger than that estimated by the other bootstrap methods. These results suggest that the TSS method may not provide stable estimates of bootstrap statistics, compared to other methods, when the statistic value of the original time series is small.

In contrast to other methods, the uPRB method’s algorithm does not require a priori tuning of parameters, such as a mean block width for the SB method. Upon providing the original time series, surrogate time series can be generated almost instantaneously. The uPRB method is characterized by its simplicity and the ability to generate surrogate time series devoid of discontinuous time points, while preserving stationarity. However, a limitation of this method is its inability to detect self-feedback (see Figure 5b). Although it accurately identifies causality among variables, some of the Confidence Intervals (CIs) have considerable width, which questions its reliability (see Figure 5a). By constraining the interval of phase randomization in Step 2, the CI width may be reduced, thereby enhancing performance; however, adjusting the degree of restriction remains arbitrary. A key distinction between the TSS and uPRB methods is that the former shifts the phases in all frequency bands simultaneously, while the latter does so for each frequency band. As the uPRB method can selectively disturb the phases corresponding to a specific frequency band, it may prove valuable for evaluating causality in the spectral domain.

For both cGC and IRF analyses, the ARSB method outperforms the other bootstrap methods, regarding detection performance. For this method, determining the VAR model order *p* is essential, which can be accomplished using the Akaike Information Criterion (AIC). As outlined in Section 3.4, there are two derivations: correlated ARSB (cARSB), which randomly shuffles the residuals across all variables synchronously, and uncorrelated ARSB (ucARSB), which shuffles the residuals independently for each variable. Our simulation study demonstrates that both derivatives yield nearly identical results. However, observational errors (artifacts) may be concurrently superimposed onto the data when analyzing actual time series, rendering ucARSB potentially more effective for canceling noise correlation among variables.

Finally, we emphasize that beyond Granger causality, as discussed in this paper, other approaches to causality estimation have been proposed; as an example, we mention Convergent Cross Mapping, which has been shown to be effective in deterministic non-linear dynamical systems [20]. Furthermore, Transfer Entropy has been defined as an extension of mutual information, representing the causality from one random variable to another [21]. Moreover, Zenil et al. [22] have developed a very general framework for model discovery, which may overcome the inherent limitations of statistical methods based on predictive models and entropy-like measures; their method is based on algorithmic probability, i.e., on decomposing observations into the most likely algorithmic generative models. Future research should investigate whether this framework can compensate for or replace the incompleteness of Granger causality.

## Figures and Tables

**Table 1:** VAR parameters for the simulation.

$A_{1}=\left(\begin{array}{ccccccccc} & 0.828 & 0 & 0 & 0 & 0 & 0 & 0 & \\ & 0.541 & 0.651 & 0 & 0 & 0 & 0 & 0 & \\ & 0.74 & 0 & 0.744 & 0 & 0 & 0 & 0 & \\ & 0 & 0 & 0.456 & 0.73 & 0.3 & 0 & 0 & \\ & 0 & 0 & 0 & -0.4 & 0.859 & 0 & 0 & \\ & 0 & 0 & 0 & 0 & 0 & 1.752 & 0 & \\ & 0 & 0 & 0 & 0 & 0 & -0.120 & 0 & \end{array}\right)$
$A_{2}=\left(\begin{array}{ccccccccc} & -0.172 & 0 & 0 & 0 & 0.17 & 0 & 0 & \\ & 0 & -0.107 & 0 & 0 & 0 & 0 & 0 & \\ & 0 & 0.238 & -0.139 & 0 & 0 & 0 & 0 & \\ & 0 & 0 & 0 & -0.134 & 0 & 0 & 0 & \\ & 0 & 0 & 0 & 0 & -0.185 & 0 & 0 & \\ & 0 & 0 & 0 & 0 & 0 & -0.810 & 0 & \\ & 0 & 0 & 0 & 0 & 0 & 0.430 & 0 & \end{array}\right)$

**Table 2:** Sensitivity and specificity of detected causality by Granger Causality and Impulse response function, according to different bootstrap methods. Numbers in parentheses indicate mean block width *w* for correlated and uncorrelated Stationary Bootstrap (cSB and ucSB, respectively).

	Granger Causality		Impulse Response	
Bootstrap Method	Sensitivity	Specificity	Sensitivity	Specificity
cSB(5)	1.0	1.0	0.94	0.93
cSB(10)	1.0	1.0	1.0	0.89
cSB(20)	1.0	1.0	1.0	0.89
cSB(40)	1.0	1.0	1.0	0.93
ucSB(5)	1.0	1.0	0.41	1.0
ucSB(10)	0.86	1.0	0.41	1.0
ucSB(20)	0.64	1.0	0.41	1.0
ucSB(40)	0.64	1.0	0.41	1.0
uPRB	0.64	1.0	0.76	0.78
TSS	0.57	1.0	0.41	1.0
cARSB	1.0	1.0	1.0	1.0
cuARSB	1.0	1.0	1.0	1.0

## Data Availability

Not applicable.
